# 522. Management of Pediatric Burns: Outcomes from an Operative-first Protocol

**DOI:** 10.1093/jbcr/irag033.219

**Published:** 2026-04-06

**Authors:** Michael J Arnold, Christopher Vakkur, Bounthavy F Homsombath, Shawn P Fagan, Martin Hardy, Zaheed Hassan, Tayseer Chowdhry, Rajiv S Sood

**Affiliations:** Medical College of Georgia, Augusta, GA; Medical College of Georgia, Augusta, GA; Joseph M. Still Burn Centers, Inc., Augusta, GA; Joseph M. Still Burn Centers, Inc., Augusta, GA; Joseph M. Still Burn Centers, Inc., Augusta, GA; Joseph M. Still Burn Centers, Inc., Augusta, GA; Burn and Reconstructive Centers of America, Augusta, GA; Burn and Reconstructive Centers of America, Augusta, GA

## Abstract

**Introduction:**

While literature often supports early surgical intervention for improved outcomes, pediatric burn care has traditionally been managed conservatively, with expectant approaches favored in many centers. Our institution employs an immediate operative protocol, using early debridement and temporary biologic coverage with cadaveric or amniotic grafts. This study describes outcomes from this operative-first strategy and evaluates its safety, efficiency, and need for subsequent interventions.

**Methods:**

We retrospectively reviewed 628 pediatric burn patients managed with early operative intervention between 2021 and 2023. Data collected included demographics, burn characteristics, type of biologic coverage, hospital course, complications, and reconstructive procedures. Outcomes were stratified by burn size and treatment course.

**Results:**

Of 628 patients, 505 had complete records for analysis. The mean age was 6.1 years, with 60% of the patients being male. Average TBSA was 6.8% (range 0.5–71.5%). Temporary biologic coverage included a cadaveric allograft in 73% of cases, with other combinations in 19.7%. Autografting was required in 74 patients (15%), most with larger burns (mean TBSA 14.6%). Of these, 18 (24%) required regrafting (mean TBSA 17%). Reconstructive procedures were necessary in 28 patients (5.5%), primarily among those with larger burn sizes (mean TBSA 20.3%). In contrast, 431 patients healed without autografting, of whom only 6 (1.3%) required reconstructive procedures. Hospital stays were brief for patients with <20% TBSA involvement (mean 1.4 days), while those with >20% TBSA had significantly longer admissions (mean 48.9 days). Only seven anesthesia-related events occurred (1.3%).

**Conclusions:**

An early operative-first protocol for pediatric burns was safe and effective, with minimal perioperative morbidity and low complication rates. The use of cadaveric or amniotic grafts reduced the need for autografting and limited secondary reconstructive procedures. Most patients required only short hospitalizations, particularly when TBSA was <20%.

**Applicability of Research to Practice:**

These findings support the feasibility of early operative management in pediatric burns, demonstrating minimized long-term morbidity, decreased need for autografting, and favorable outcomes while maintaining a low risk of perioperative complications.

**Funding for the study:**

N/A.

